# Endothelin-1 induces connective tissue growth factor expression in human lung fibroblasts by disrupting HDAC2/Sin3A/MeCP2 corepressor complex

**DOI:** 10.1186/s12929-023-00931-5

**Published:** 2023-06-14

**Authors:** Hung-Sheng Hua, Heng-Ching Wen, Hong-Sheng Lee, Chih-Ming Weng, Fara Silvia Yuliani, Han-Pin Kuo, Bing-Chang Chen, Chien-Huang Lin

**Affiliations:** 1 Graduate Institute of Medical Sciences, College of Medicine, Taipei Medical University, 250 Wu-Hsing Street, Taipei, 110 Taiwan; 2 School of Respiratory Therapy, College of Medicine, Taipei Medical University, 250 Wu-Hsing Street, Taipei, 110 Taiwan; 3 Division of Thoracic Medicine, Department of Internal Medicine, School of Medicine, College of Medicine, Taipei Medical University, Taipei, Taiwan; 4 Research Center of Thoracic Medicine, Taipei Medical University, Taipei, Taiwan; 5 Department of Thoracic Medicine, Taipei Medical University Hospital, Taipei, Taiwan; 6 Department of Pharmacology and Therapy, Faculty of Medicine, Public Health and Nursing, Universitas Gadjah Mada, Yogyakarta, Indonesia

**Keywords:** HDAC2, Sin3A, MeCP2, ET-1, CTGF, Airway fibrosis, Lung fibroblasts

## Abstract

**Background:**

Reduction of histone deacetylase (HDAC) 2 expression and activity may contribute to amplified inflammation in patients with severe asthma. Connective tissue growth factor (CTGF) is a key mediator of airway fibrosis in severe asthma. However, the role of the HDAC2/Sin3A/methyl-CpG-binding protein (MeCP) 2 corepressor complex in the regulation of CTGF expression in lung fibroblasts remains unclear.

**Methods:**

The role of the HDAC2/Sin3A/MeCP2 corepressor complex in endothelin (ET)-1-stimulated CTGF production in human lung fibroblasts (WI-38) was investigated. We also evaluated the expression of HDAC2, Sin3A and MeCP2 in the lung of ovalbumin-induced airway fibrosis model.

**Results:**

HDAC2 suppressed ET-1-induced CTGF expression in WI-38 cells. ET-1 treatment reduced HDAC2 activity and increased H3 acetylation in a time-dependent manner. Furthermore, overexpression of HDAC2 inhibited ET-1-induced H3 acetylation. Inhibition of c-Jun N-terminal kinase, extracellular signal-regulated kinase, or p38 attenuated ET-1-induced H3 acetylation by suppressing HDAC2 phosphorylation and reducing HDAC2 activity. Overexpression of both Sin3A and MeCP2 attenuated ET-1-induced CTGF expression and H3 acetylation. ET-1 induced the disruption of the HDAC2/Sin3A/MeCP2 corepressor complex and then prompted the dissociation of HDAC2, Sin3A, and MeCP2 from the CTGF promoter region. Overexpression of HDAC2, Sin3A, or MeCP2 attenuated ET-1-stimulated AP-1-luciferase activity. Moreover, Sin3A- or MeCP2-suppressed ET-1-induced H3 acetylation and AP-1-luciferase activity were reversed by transfection of HDAC2 siRNA. In an ovalbumin-induced airway fibrosis model, the protein levels of HDAC2 and Sin3A were lower than in the control group; however, no significant difference in MeCP2 expression was observed. The ratio of phospho-HDAC2/HDAC2 and H3 acetylation in the lung tissue were higher in this model than in the control group. Overall, without stimulation, the HDAC2/Sin3A/MeCP2 corepressor complex inhibits CTGF expression by regulating H3 deacetylation in the CTGF promoter region in human lung fibroblasts. With ET-1 stimulation, the HDAC2/Sin3A/MeCP2 corepressor complex is disrupted and dissociated from the CTGF promoter region; this is followed by AP-1 activation and the eventual initiation of CTGF production.

**Conclusions:**

The HDAC2/Sin3A/MeCP2 corepressor complex is an endogenous inhibitor of CTGF in lung fibroblasts. Additionally, HDAC2 and Sin3A may be of greater importance than MeCP2 in the pathogenesis of airway fibrosis.

## Background

Lung fibrosis is a heterogeneous respiratory disease with a high mortality rate. It is characterized by scar formation in lung tissue, which causes lung architecture destruction, leading to death [54]. Subepithelial fibrosis is characterized by extensive deposition of extracellular matrix (ECM), including collagens, in the airway wall, which may lead to progressive airflow obstruction [6, 50]. The pathogenesis of lung fibrosis primarily involves fibroblast differentiation and proliferation [55]. In pathological fibrosis, fibroblasts differentiate into myofibroblasts, which excessively synthesize ECM [15]. Fibroblast differentiation is regulated by various profibrotic factors, such as connective tissue growth factor (CTGF) [56], transforming growth factor (TGF)-β1 [40], and thrombin [5].

CTGF, formerly known as CCN2, mediates various biological processes, such as ECM production and tissue modeling, as well as cell adhesion, migration, proliferation, and differentiation [34]. A study indicated that CTGF is an essential downstream mediator for TGF-β1-induced ECM production and myofibroblast differentiation in Graves’ orbital fibroblast [48]. In bleomycin-induced lung fibrosis, CTGF contributes to lung fibrosis by promoting type I collagen production in lung fibroblasts [43]. CTGF plays a central mediating role in tissue fibrosis [38]. In our previous studies, we have demonstrated that CTGF production in human lung fibroblasts is stimulated by several profibrotic factors, such as CXC motif chemokine ligand 12, thrombin, hypoxia, and endothelin (ET)-1 [9, 11, 37, 53].

Elevated expression of ET-1 by the bronchial epithelium is associated with airflow obstruction and airway remodeling, and it is often observed in severe refractory asthma [42]. In our previous studies, we have observed the following: first, the ET_A_ receptor (ET_A_R)-dependent pathway mediates CTGF expression, which prompts fibrocyte differentiation into myofibroblasts in chronic obstructive asthma [52]; second, ET-1 stimulates CTGF production through c-Jun N-terminal kinase (JNK)/activator protein (AP)-1 activation in lung fibroblasts [53]; third, ET-1 activates HDAC7 and p300, thereby leading to the initiation of AP-1 transcriptional activity and eventually the promotion of CTGF production in lung fibroblasts [22].

Epigenetic modifications of histone play a pivotal role in both the regulation of gene expression and the development of various diseases [8]. Histone acetylation and deacetylation are involved in the pathogenesis of asthma and chronic obstructive pulmonary disease (COPD) through the regulation of inflammatory genes [3]. Histone acetylation and deacetylation are controlled by histone acetyltransferases and histone deacetylase (HDAC). Several studies have suggested that a reduction in HDAC2 expression and activity may contribute to amplified inflammation in individuals with severe asthma or COPD [13, 24]. In steroid-resistant asthma, glucocorticoid receptor β contributes to steroid resistance by inhibiting HDAC2 expression [35]. HDAC2 reduction plays a crucial role in glucocorticoid insensitivity by repressing NF-κB-mediated gene expression [25]. Passive smoking impairs HDAC2 function, which may contribute to corticosteroid-insensitive inflammation in children with severe asthma [32].

HDAC2 is known to form a corepressor complex with a large scaffold protein, Sin3A, to regulate gene transcription [46]. The Sin3A/HDAC2 complex serves as a negative regulator of the inflammatory gene program in lipopolysaccharide-activated human macrophages [26]. A study indicated that methyl-CpG-binding protein 2 (MeCP2) recruits HDAC2 and Sin3A to promote the deacetylation of histone tails, which results in gene silencing [28]. The methylation of DNA results in the recruitment of HDAC-containing complexes through MeCP2 to strengthen histone deacetylation, which then leads to reinforced epigenetic silencing of the COX2 gene in fibroblasts from patients with idiopathic pulmonary fibrosis [14]. However, the role of the HDAC2/Sin3A/MeCP2 corepressor complex in ET-1-stimulated CTGF expression remains unclear.

In this study, we evaluated the roles of HDAC2, Sin3A, and MeCP2 in ET-1-stimulated CTGF expression in lung fibroblasts. We observed that the HDAC2/Sin3A/MeCP2 corepressor complex is an endogenous inhibitor of CTGF expression. ET-1 caused the disruption of the HDAC2/Sin3A/MeCP2 corepressor complex, thereby reducing HDAC2 activity and increasing histone H3 acetylation; this was followed by AP-1 recruitment to the CTGF promoter, which in turn promoted CTGF expression. Moreover, we observed a reduction in HDAC2 and Sin3A, but not MeCP2, in the lung of the ovalbumin (OVA)-induced airway fibrosis model, indicating that HDAC2 and Sin3A might play a greater role than MeCP2 in the pathogenesis of airway fibrosis.

## Materials and methods

### Materials

WI-38 normal human embryonic lung fibroblast cell lines (ATCC CCL-75) were purchased from American Type Culture Collection (Manassas, VA, USA). Recombinant human ET-1 was obtained from Bachem Americas (Torrance, CA, USA). Minimum essential medium (MEM), Lipofectamine 3000 reagent, and fetal bovine serum (FBS) were purchased from Invitrogen Life Technologies (Carlsbad, CA, USA). The chromatin immunoprecipitation (ChIP) assay kit was obtained from Upstate Biotechnology (Lake Placid, NY, USA). 2X Tools Tag PCR MasterMix with loading dye was obtained from BIOTOOLS (BIOTOOLS, Taipei, Taiwan). All materials for Western blots were acquired from Bio-Rad (Hercules, CA, USA). Antibodies specific for HDAC2, CTGF, anti-mouse, and anti-rabbit immunoglobulin G (IgG)-conjugated horseradish peroxidase were obtained from Santa Cruz Biotechnology (Santa Cruz, CA, USA). Antibodies specific for Sin3A, MeCP2, and GFP-tag, were purchased from Cell Signaling Technology (Danvers, MA, USA), and the anti-α-tubulin antibody was purchased from Transduction Laboratories (Lexington, KY, USA). Antibodies specific for H3, acetyl-H3, phospho-HDAC2 (S394), HDAC7, and HA-tag were obtained from Abcam (Cambridge, MA, USA). Anti-rabbit IgG (Alexa Fluor® 555) was obtained from Abcam. Alexa Fluor® 488-conjugated HDAC2 antibody and Alexa Fluor® 647-conjugated phospho-HDAC2 (S394) antibody were purchased from Bioss (Woburn, MA, USA). HDAC2 activity assay kit were purchased from Abcam. HDAC2-HA, Sin3A-HA, MeCP2-HA, and HDAC7-GFP plasmid were purchased from GeneCopoeia (Rockville, MD, USA). pBK-CMV-Lac Z (*LacZ*) was obtained from Dr. W-W. Lin (National Taiwan University, Taipei, Taiwan). pAP-1-Luc *cis*-Reporter plasmid was obtained from Stratagene (Santa Clara, CA, USA). The luciferase assay system was procured from Promega (Madison, WI, USA), OVA was bought from MilliporeSigma (Burlington, MA, USA), aluminum hydroxide was obtained from Thermo Fisher Scientific (Waltham, MA, USA), and all other chemicals were acquired from MilliporeSigma.

### Cell culture

WI-38 cells at passages 22–30 were used to conduct experiments. Cells were grown in a CO_2_ incubator at 37 °C. MEM supplemented with 10% FBS was used for cell cultures. For transfection and immunoblotting, cells were seeded into 6-cm dishes. For Co-IP and ChIP assays, cells were transferred into 10-cm dishes. For transfection and luciferase assays, cells were seeded into 12-well plates.

### Transfection and luciferase reporter assays

WI-38 cells were seeded into 12-well plates (5 × 10^4^ cells/well). Plasmids of AP-1-Luc, *Lac Z*, HDAC2-HA, Sin3A-HA, MeCP2-HA, or HDAC2 siRNA were transfected into WI-38 cells by using Lipofectamine 3000. After transfection for 6 h, the culture medium was replaced with serum-free medium, and the cells were incubated overnight. The WI-38 cells were then treated with ET-1 for 16 h; subsequently, their luciferase activity was assayed. The level of *LacZ* was used to normalize luciferase activity.

### Western blot analysis

Cell lysates were separated through sodium dodecyl sulfate polyacrylamide gel electrophoresis and then transferred to a polyvinylidene difluoride membrane. The membrane was immersed in bovine serum albumin solution (1%) for 1 h at room temperature to block nonspecific antigens on the membrane and was subsequently incubated with primary antibodies at 4 °C overnight. Antibodies specific for HDAC2, CTGF, phospho-HDAC2 (S394), Sin3A, MeCP2, acetyl-H3, H3, HDAC7, GFP-tag, HA-tag, or α-tubulin were used to target the proteins. After incubation with primary antibodies, the membrane was incubated with secondary antibody for 1 h. The blots were displayed using enhanced chemiluminescence reagents. Quantitative data were obtained using a scientific imaging system (Kodak, Rochester, NY, USA).

### Coimmunoprecipitation

Cell lysates were collected and centrifuged. The supernatants were incubated with protein A/G beads and antibodies specific for HDAC2, Sin3A, or MeCP2 at 4 °C overnight for coimmunoprecipitation. The interaction between proteins was then assayed using Western blotting.

### ChIP assay

The samples were sonicated, centrifuged at 15,000×*g* at 4 °C for 10 min, and subsequently immunoprecipitated with HDAC2, Sin3A, MeCP2, acetyl-H3, or rabbit IgG antibody and protein A/G beads overnight. After immunoprecipitation, DNA was purified to amplify the AP-1 binding site by using polymerase chain reaction (PCR). The primer sequences were as follows: 5′-CGT CCC TTG TCC TTG CCT AT-3′ (sense) and 5′-GCT CGA CCT CAC ACG GTC GA-3′ (antisense). The PCR involved 45 cycles of amplification at 95 °C for 30 s, 52 °C for 60 s, and 72 °C for 30 s.

### Sensitization and antigen challenge of an asthmatic animal model

Female C57BL/6 mice (BioLASCO, Taipei, Taiwan) aged 7 weeks were used in the experiments. They were divided into two groups (*n* = 10 per group): control (phosphate-buffered saline [PBS]) and OVA treatment. The mice were immunized through subcutaneous injections of 50 μg of OVA adsorbed on 4 mg of aluminum hydroxide in 200 μL of PBS on days 1, 8, and 15. OVA challenges (20 μg/50 μL in PBS) were initiated on day 28 and were repeated twice a week for 8 weeks in an ultrasonic nebulizer chamber. The control mice were treated similarly with PBS but without OVA. The mice were sacrificed at 12 weeks. All animal experimental protocols were approved by the Taipei Medical University Institutional Animal Care and Use Committee (LAC-101-0243).

### Immunofluorescence (IF) staining

The expression of phospho-HDAC2, HDAC2, and Sin3A in the lung of OVA-treated mice and in ET-1-stimulated WI-38 cells were visualized by immunofluorescent staining. The paraffin-embedded lung tissue sections of mice were processed for antigen retrieval. WI-38 cells were stimulated with ET-1 for 10 min and then fixed with 4% formaldehyde for 10 min at 37 °C. The lung tissue sections or WI-38 cells were blocked with 5 mg/mL bovine serum albumin for 1 h. Alexa Fluor® 488-conjugated HDAC2 antibody and Alexa Fluor® 647-conjugated phospho-HDAC2 (S394) antibody were stained respectively for 2 h. Sin3A antibody was stained for 2 h and incubated with Alexa Fluor® 555-conjugated secondary antibody for another 1 h. Each slide was stained with DAPI to visualize nuclei and as a live cell marker. The immunofluorescence staining slides were examined through a fluorescence microscope.

### Statistical analysis

At least three independent experiments were conducted. Data are presented as mean ± standard error of the mean. A one-way analysis of variance was conducted followed by Dunnett’s test. In all cases, *p* < 0.05 was considered statistically significant. The calculation process of inhibition rate is as follows: the percentage of control = A; the percentage of ET-1 stimulation group = B; the percentage of inhibitors with ET-1 stimulation = C; inhibition rate = 1 − [(C − A)/(B − A)].

## Results

### HDAC2 suppressed ET-1-induced CTGF production

**Fig. 1:**
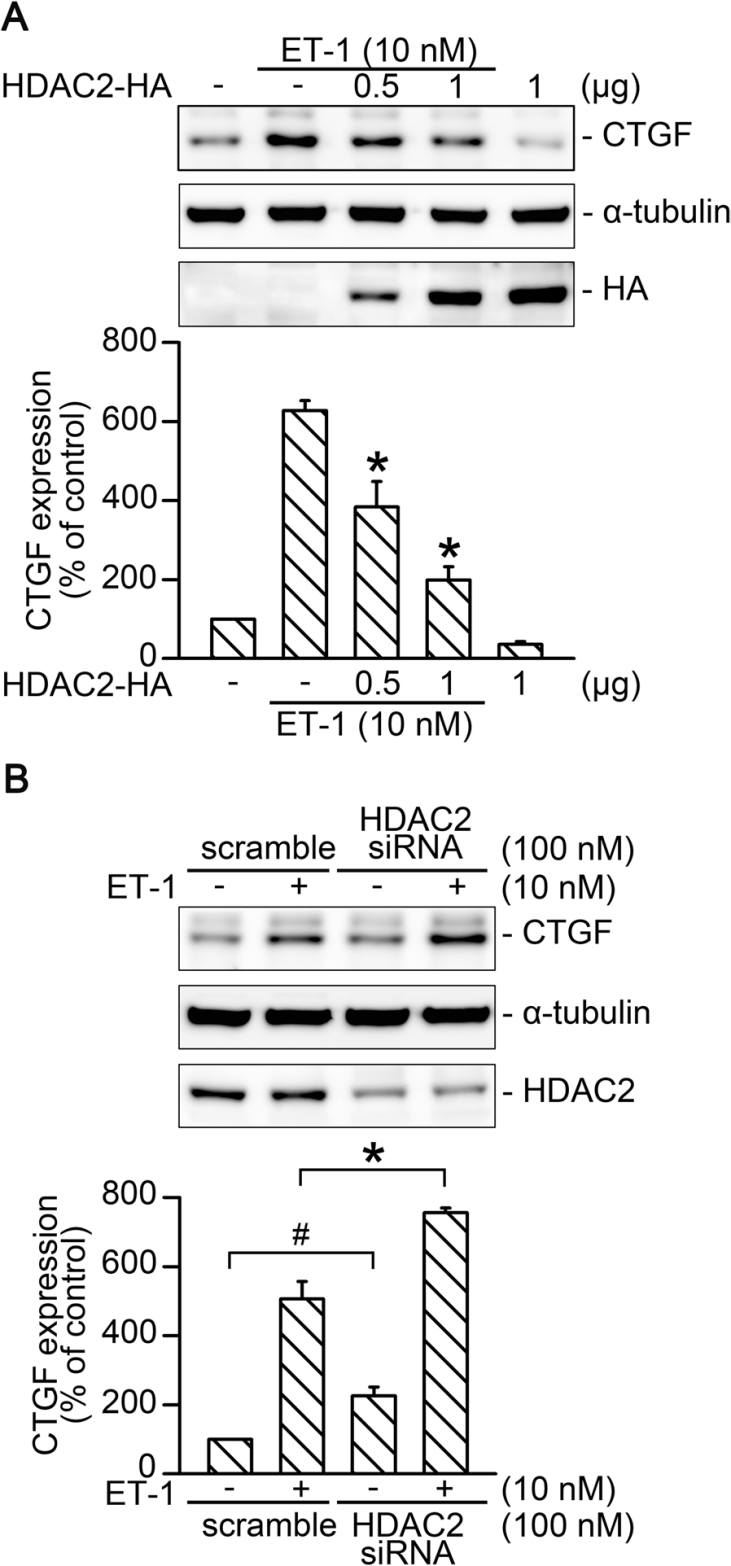
HDAC2 negatively regulated ET-1-induced CTGF production in WI-38 cells. **A** Cells were transfected with either 0.5 or 1 μg of HDAC2-HA plasmid or pcDNA for 24 h and were then treated with ET-1 for 2 h. The CTGF protein level was analyzed by Western blotting. Antibody specific for α-tubulin was applied to determine loading control. HA-tag was used to confirm the overexpression of HDAC2. Bars indicate the mean ± standard error of the mean (SEM; *n* = 4). *p < 0.05 versus the group with ET-1 stimulation. **B** Cells were transfected with HDAC2 siRNA for 24 h and treated with ET-1 for 2 h; subsequently, the CTGF protein level was analyzed. Bars indicate the mean ± SEM (*n* = 5). ^#^*p* < 0.05 versus control, *p < 0.05 versus ET-1 treatment

Reduced HDAC2 activity is associated with the elevation of inflammatory gene expression in patients with severe asthma [2]. In this study, we evaluated the role of HDAC2 in ET-1-stimulated CTGF expression in WI-38 cells. HDAC2 overexpression (0.5 and 1 μg) attenuated ET-1-stimulated CTGF production in a dose-dependent manner (*n* = 4; Fig. 1A). Without ET-1 stimulation, transfection of HDAC2 small interfering RNA (siRNA; 100 nM) increased CTGF protein level in WI-38 cells by 226% ± 25% (*n* = 5; Fig. 1B). With ET-1 stimulation, transfection of HDAC2 siRNA enhanced the CTGF protein level by 157% ± 20% (*n* = 5; Fig. 1B). Thus, without stimulation, HDAC2 was an endogenous inhibitor of CTGF expression. With ET-1 stimulation, HDAC2 negatively regulated ET-1-stimulated CTGF production in WI-38 cells.

### ET-1 induced histone H3 acetylation through a reduction in HDAC2 activity

**Fig. 2:**
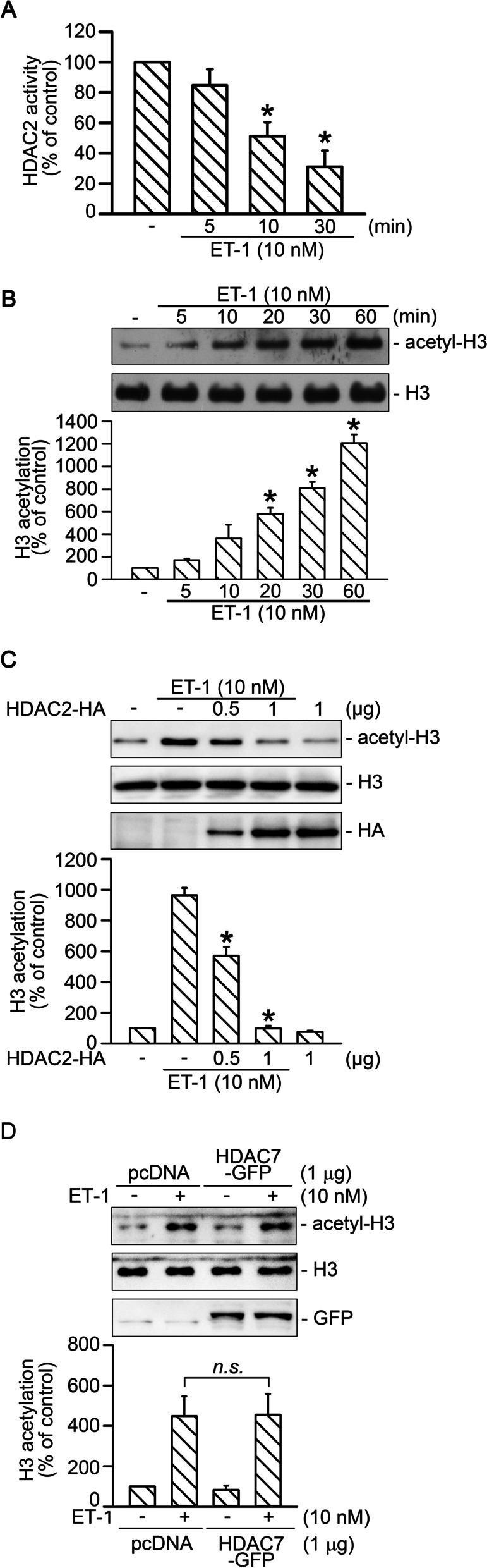
ET-1 induced a decrease in HDAC2 activity and acetylation of H3. **A** Cells were stimulated with ET-1 for 5, 10, or 30 min. The cell lysates were immunoprecipitated with antibody specific for HDAC2, and then HDAC activity was detected. Bars indicate values of the mean ± SEM (*n* = 3). *p < 0.05 versus control group. **B** Cells were stimulated with ET-1 for 5, 10, 20, 30, or 60 min, and then the acetylation of histone H3 was evaluated using Western blotting. H3 was used as a loading control. Bars indicate mean ± SEM (*n* = 4). *p < 0.05 versus control group. **C** Cells were transfected with either 0.5 or 1 μg of HDAC2-HA plasmid for 24 h and then treated with ET-1 for 20 min. Cells were subsequently lysed and immunoblotted with antibodies specific for histone H3, acetyl-H3, or HA. Bars indicate values of the mean ± SEM (*n* = 4). *p < 0.05 versus ET-1-treated group. **D** Cells were transfected with 1 μg of HDAC7-GFP plasmid for 24 h and then treated with ET-1 for 20 min. Cells were subsequently lysed and immunoblotted with antibodies specific for histone H3, acetyl-H3, or GFP. Bars indicate values of the mean ± SEM (*n* = 5)

CTGF expression is regulated through the epigenetic modification of DNA, such as histone acetylation [12]. In our previous study, HDAC7 regulated ET-1-induced CTGF expression through the activation of AP-1 in WI-38 cells [22]. In this study, we evaluated the role of HDAC2 and HDAC7 in ET-1-induced histone H3 acetylation. When cells were treated with ET-1 for 5–30 min, the HDAC2 activity decreased in a time-dependent manner (*n* = 3; Fig. 2A). By contrast, treating the cells with ET-1 for 5–60 min increased H3 acetylation in a time-dependent manner (*n* = 4; Fig. 2B). Further, HDAC2 overexpression attenuated ET-1-stimulated H3 acetylation in a dose-dependent manner (*n* = 4; Fig. 2C). However, overexpression of HDAC7 did not affect ET-1-induced H3 acetylation (*n* = 5; Fig. 2D). Thus, ET-1 induced H3 acetylation by suppressing HDAC2 activity, but not HDAC7 activation.

### JNK, extracellular signal-regulated kinase, and p38 were involved in ET-1-induced H3 acetylation through the regulation of HDAC2 phosphorylation and HDAC2 activity

**Fig. 3:**
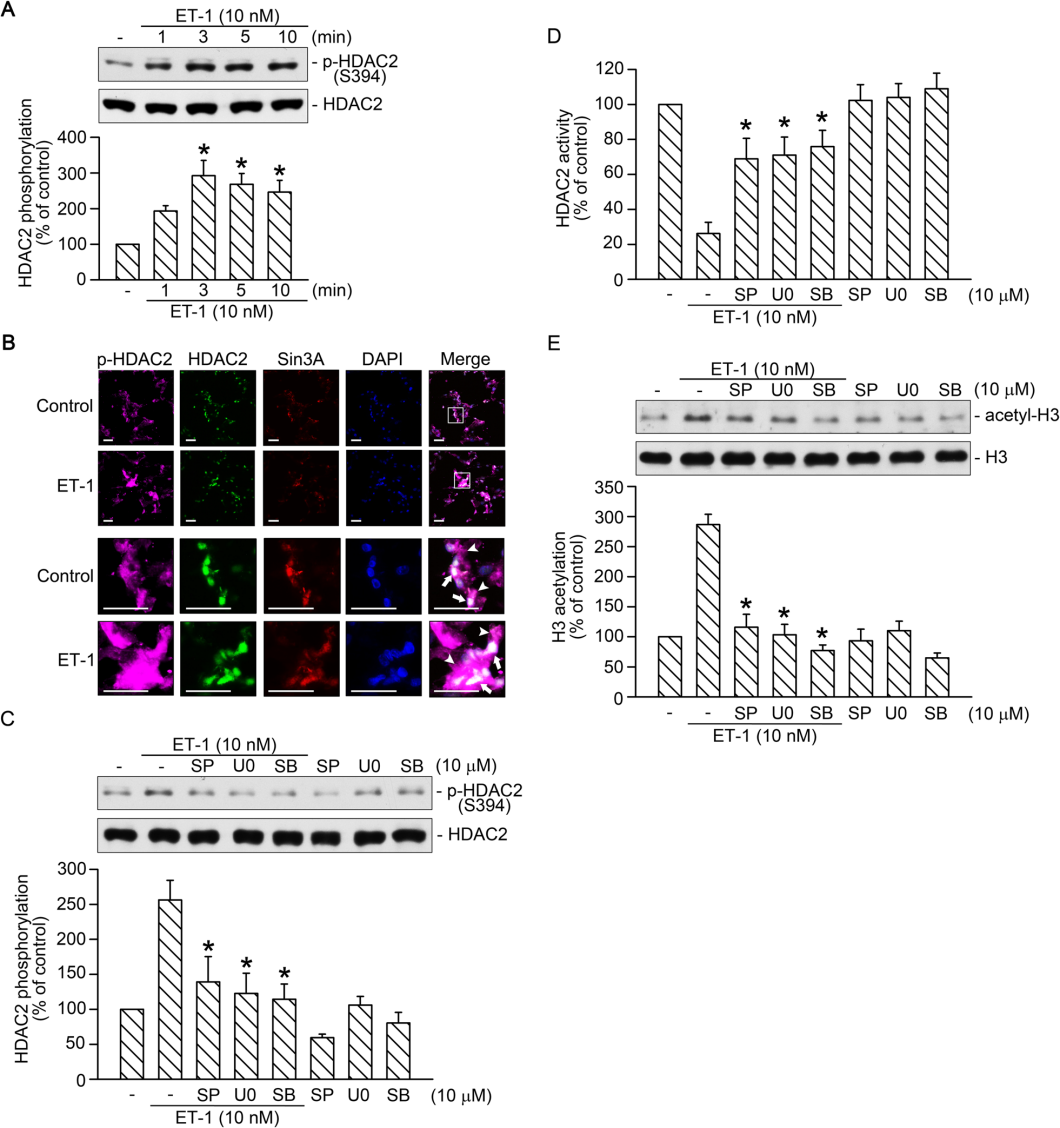
JNK, ERK, and p38 were involved in ET-1-induced H3 acetylation through the regulation of HDAC2 phosphorylation and HDAC2 activity. **A** Cells were stimulated with ET-1 for 1, 3, 5, or 10 min and then lysed and immunoblotted with antibodies specific for HDAC2 or phospho-HDAC2 (S394). Bars indicate values of the mean ± SEM (*n* = 3). *p < 0.05 versus control group. **B** After ET-1 stimulation for 10 min, WI-38 cells were immunodetected with antibodies specific for phosphor-HDAC2 (purple), HDAC2 (green), and Sin3A (red); nuclei were detected with DAPI (blue). The nuclei is labeled by a white arrow. The cytosol is labeled by a white arrowhead. Bar, 50 μm. **C** Cells were pretreated with 10 μM of SP600125, U0126, or SB203580 or an equivalent vehicle control (dimethyl sulfoxide [DMSO]) for 30 min and then treated with ET-1 for 3 min. The cells were then lysed and immunoblotted with antibodies specific for HDAC2 or phospho-HDAC2 (S394). Bars indicate values of the mean ± SEM (*n* = 4). *p < 0.05 versus ET-1 stimulation. **D** Cells were pretreated with 10 μM SP600125, 10 μM U0126, or 10 μM SB203580 or DMSO for 30 min and then stimulated with ET-1 for 20 min. The cell lysates were immunoprecipitated with antibodies specific for HDAC2, and then HDAC activity was detected. Bars indicate values of the mean ± SEM (*n* = 4). *p < 0.05, versus ET-1 stimulation. **E** Cells were pretreated with 10 μM of SP600125, U0126, or SB203580 or DMSO for 30 min and then treated with ET-1 for 20 min. The cells were then lysed and immunoblotted with antibodies specific for histone H3 or acetyl-H3. Bars indicate values of the mean ± SEM (*n* = 5). *p < 0.05 versus ET-1stimulation

HDAC2 phosphorylation at serine sites S394, S422, and S424 negatively regulates its deacetylase activity [1]. Moreover, the phosphorylation of HDAC2 is crucial for the dissociation of HDAC2 and Sin3A [30]. In this study, we found that incubation of cells with ET-1 for 1–10 min stimulated HDAC2 phosphorylation at S394 in a time-dependent manner (*n* = 3; Fig. 3A). In addition, we identify the changes and location of phospho-HDAC2, HDAC2, and Sin3A in WI-38 cells by IF staining. Without ET-1 stimulation, phospho-HDAC2 was expressed in both nuclei and cytosol; meanwhile, HDAC2 and Sin3A were expressed mainly in nuclei (Fig. 3B). With ET-1 stimulation, the level of phospho-HDAC2 was increased in both nuclei and cytosol compared with control (Fig. 3B). However, no difference was observed in the level of HDAC2 and Sin3A (Fig. 3B). Previous studies suggested that HDAC2 can be phosphorylated by JNK [39] and possibly by other members of the mitogen-activated protein kinase (MAPK) family, such as extracellular signal-regulated kinase (ERK) and p38 [44]. In this study, we investigated whether MAPKs mediate ET-1-induced HDAC2 phosphorylation and subsequent H3 acetylation. We found that pretreatment of cells with SP600125 (JNK inhibitor), U0126 (ERK inhibitor), or SB203580 (p38 inhibitor) attenuated ET-1-induced HDAC2 phosphorylation by 77% ± 25%, 89% ± 21%, and 96% ± 13% (*n* = 4; Fig. 3C) and mitigated ET-1-induced reduction in HDAC2 activity by 59% ± 17%, 62% ± 14%, and 69% ± 13%, respectively (*n* = 4; Fig. 3D). Furthermore, pretreatment of cells with SP600125, U0126, or SB203580 attenuated ET-1-induced H3 acetylation by 78% ± 14%, 92% ± 9%, and 111% ± 7%, respectively (*n* = 5; Fig. 3E). Thus, the ET-1-induced reduction in HDAC2 activity and increase in H3 acetylation were mediated by the JNK, ERK, and p38 pathways.

### Sin3A and MeCP2 were negative regulators in ET-1-induced CTGF expression

**Fig. 4:**
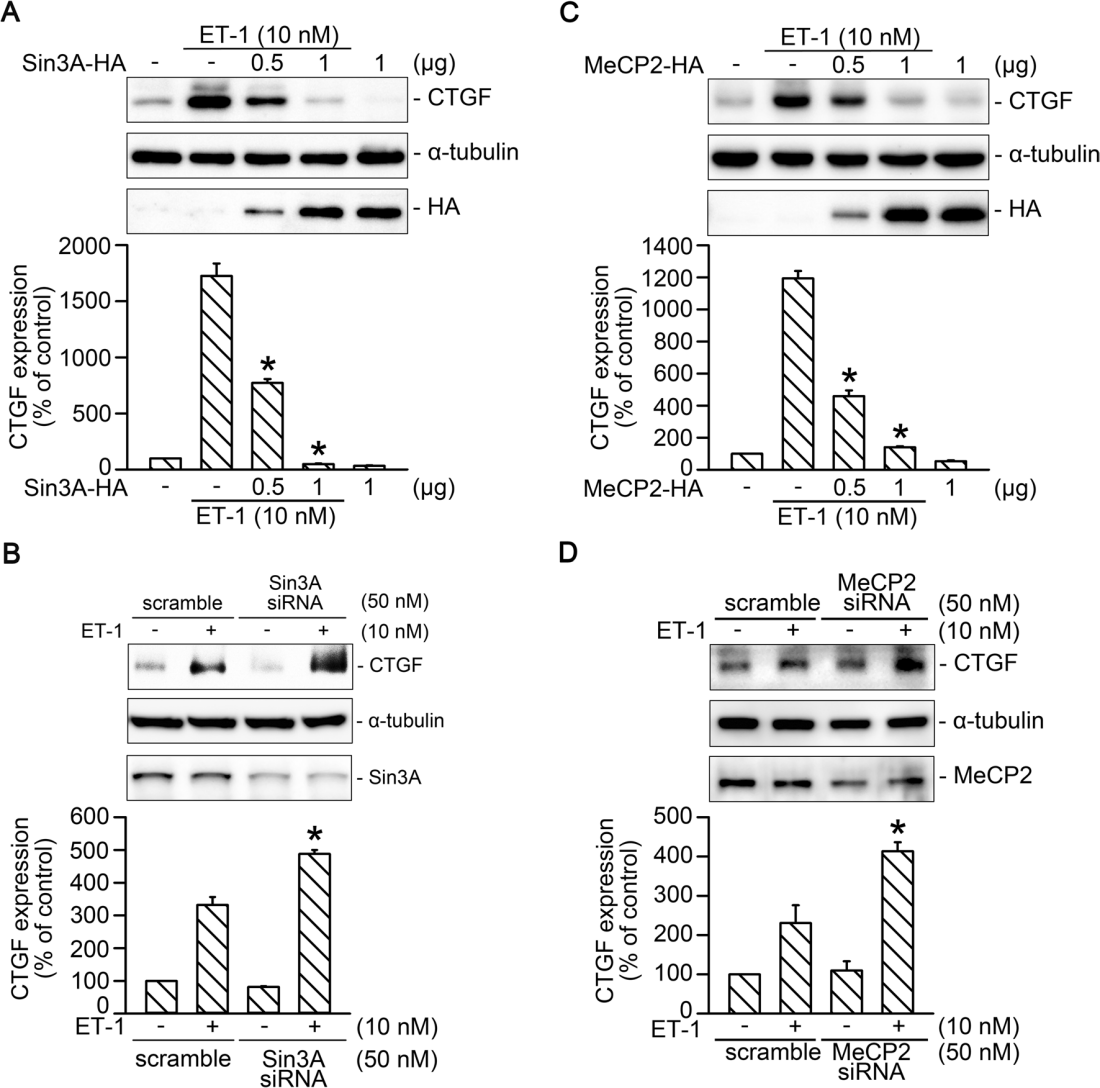
Sin3A and MeCP2 negatively regulated ET-1-induced CTGF production. **A** Cells were transfected with either 0.5 or 1 μg of Sin3A-HA plasmid or pcDNA for 24 h and then treated with ET-1 for 2 h. The protein levels of CTGF and HA-tag were evaluated through Western blotting. Bars indicate values of the mean ± SEM (*n* = 4). *p < 0.05 versus the ET-1-treated group. **B** Sin3A was knocked down through transfection with siRNA for 24 h. After 2 h of stimulation with ET-1, the CTGF protein level was evaluated using Western blotting. Bars indicate values of the mean ± SEM (*n* = 3). *p < 0.05 versus the ET-1-treated group. **C** Cells were transfected with either 0.5 or 1 μg of MeCP2-HA plasmid or pcDNA for 24 h and then treated with ET-1 for 2 h. The protein levels of CTGF and HA-tag were evaluated using Western blotting. Bars indicate values of the mean ± SEM (*n* = 6). *p < 0.05 versus the ET-1 stimulation group. **D** MeCP2 was knocked down through transfection with siRNA for 24 h. After 2 h of stimulation with ET-1, the CTGF protein level was examined using Western blotting. Bars indicate values of the mean ± SEM (*n* = 4). *p < 0.05 versus the ET-1-treated group

MeCP2 recruits HDAC1, HDAC2, and Sin3A to promote the deacetylation of histone tails, thus resulting in gene silencing [28, 31]. In this study, we examined the role of Sin3A and MeCP2 in ET-1-induced CTGF production. Our results revealed that Sin3A overexpression (0.5 and 1 μg) attenuated ET-1-stimulated CTGF production in a dose-dependent manner (*n* = 4; Fig. 4A). Transfection of Sin3A siRNA (50 nM) into WI-38 cells enhanced ET-1-stimulated CTGF production by 148% ± 10% (*n* = 3; Fig. 4B). Further, overexpression of MeCP2 attenuated ET-1-stimulated CTGF expression in a dose-dependent manner (*n* = 6; Fig. 4C). Transfection of MeCP2 siRNA (50 nM) enhanced ET-1-stimulated CTGF expression by 192% ± 39% (*n* = 4; Fig. 4D). Thus, Sin3A and MeCP2 negatively regulated ET-1-stimulated CTGF production in WI-38 cells.

### HDAC2 participated in the regulation of Sin3A- or MeCP2-suppressed H3 acetylation on CTGF promoter in ET-1-stimulated WI-38 cells

**Fig. 5:**
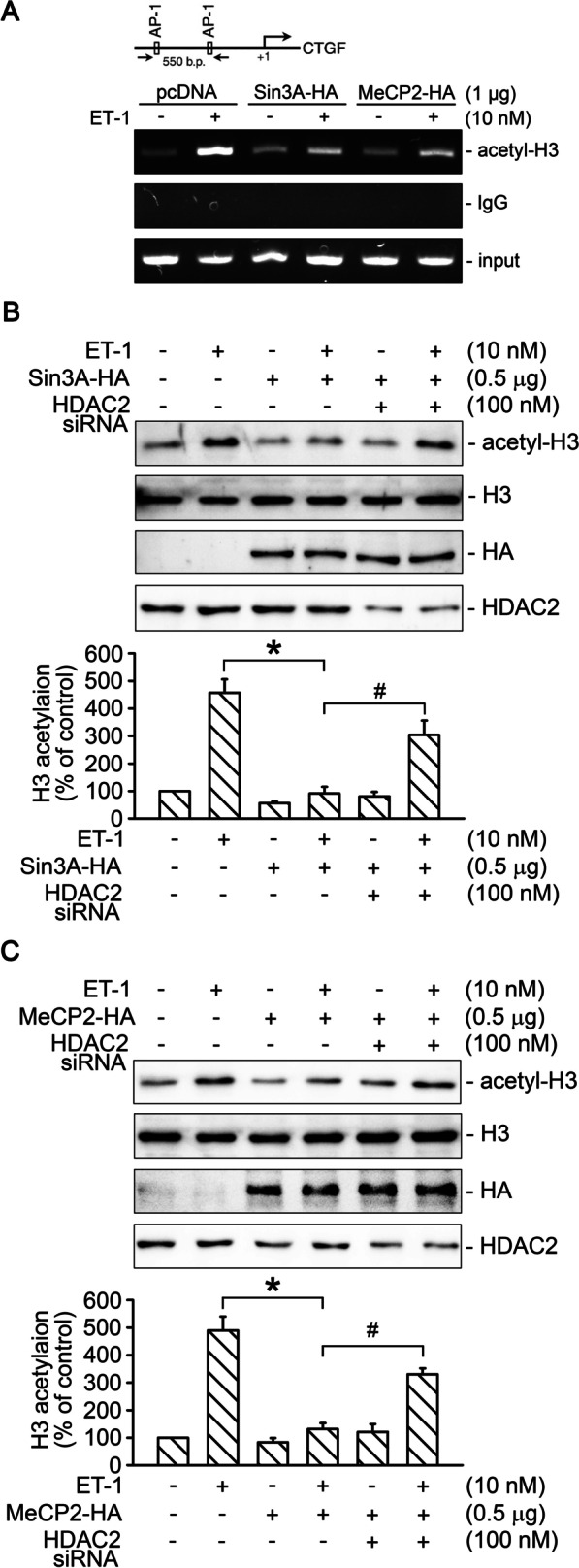
HDAC2 participated in the regulation of Sin3A- or MeCP2-suppressed H3 acetylation on CTGF promoter in ET-1-stimulated WI-38 cells. **A** Schematic of the 550-bp ChIP primer located on the CTGF promoter. Cells were transfected with either Sin3A-HA (1 μg) or MeCP2-HA plasmid (1 μg) for 24 h and then stimulated with ET-1 for 20 min, which was followed by ChIP assay. Nonimmune IgG was used as a negative control. Equal amounts of the soluble cross-linked chromatin present in each PCR were checked by the input (*n* = 3). **B** Cells were transfected with Sin3A-HA plasmid (0.5 μg) or co-transfected with Sin3A-HA plasmid (0.5 μg) and HDAC2 siRNA (100 nM) for 24 h and then treated with ET-1 for 20 min. Cells were then lysed and immunoblotted with antibodies specific for histone H3, acetyl-H3, HDAC2, or HA. Bars indicate values of the mean ± SEM (*n* = 5). *p < 0.05 versus ET-1-treated cells, ^#^*p* < 0.05 versus Sin3A-transfected cells with ET-1 stimulation. **C** Cells were transfected with MeCP2-HA plasmid (0.5 μg) or co-transfected with MeCP2-HA plasmid (0.5 μg) and HDAC2 siRNA (100 nM) for 24 h and then treated with ET-1 for 20 min. Cells were subsequently lysed and immunoblotted with antibodies specific for histone H3, acetyl-H3, HDAC2, or HA. Bars indicate values of the mean ± SEM (*n* = 5). *p < 0.05 versus ET-1-treated cells, ^#^*p* < 0.05 versus MeCP2-transfected cells with ET-1 stimulation

In order to evaluate the role of Sin3A and MeCP2 in the regulation of H3 acetylation on the CTGF promoter region, the ChIP assay was conducted. Overexpression of Sin3A or MeCP2 both suppressed ET-1-indeuced H3 acetylation on CTGF promoter region (*n* = 3; Fig. 5A). In addition, we investigated if suppression of ET-1-stimulated H3 acetylation by overexpression of Sin3A or MeCP2 was dependent on the presence of HDAC2. We found that overexpression of Sin3A attenuated ET-1-stimulated H3 acetylation by 105% ± 6%, and this response was significantly recovered by transfection of HDAC2 siRNA (*n* = 5; Fig. 5B). Moreover, MeCP2 overexpression reduced ET-1-stimulated H3 acetylation by 95% ± 6%, which this response was significantly reversed by transfection of HDAC2 siRNA (*n* = 5; Fig. 5C). Thus, Sin3A- or MeCP2-suppressed ET-1-induced H3 acetylation on CTGF prompter region was dependent on the presence of HDAC2 in WI-38 cells.

### ET-1 caused the disruption of HDAC2, Sin3A, and MeCP2 corepressor complexes and their subsequent dissociation from CTGF promoter

**Fig. 6:**
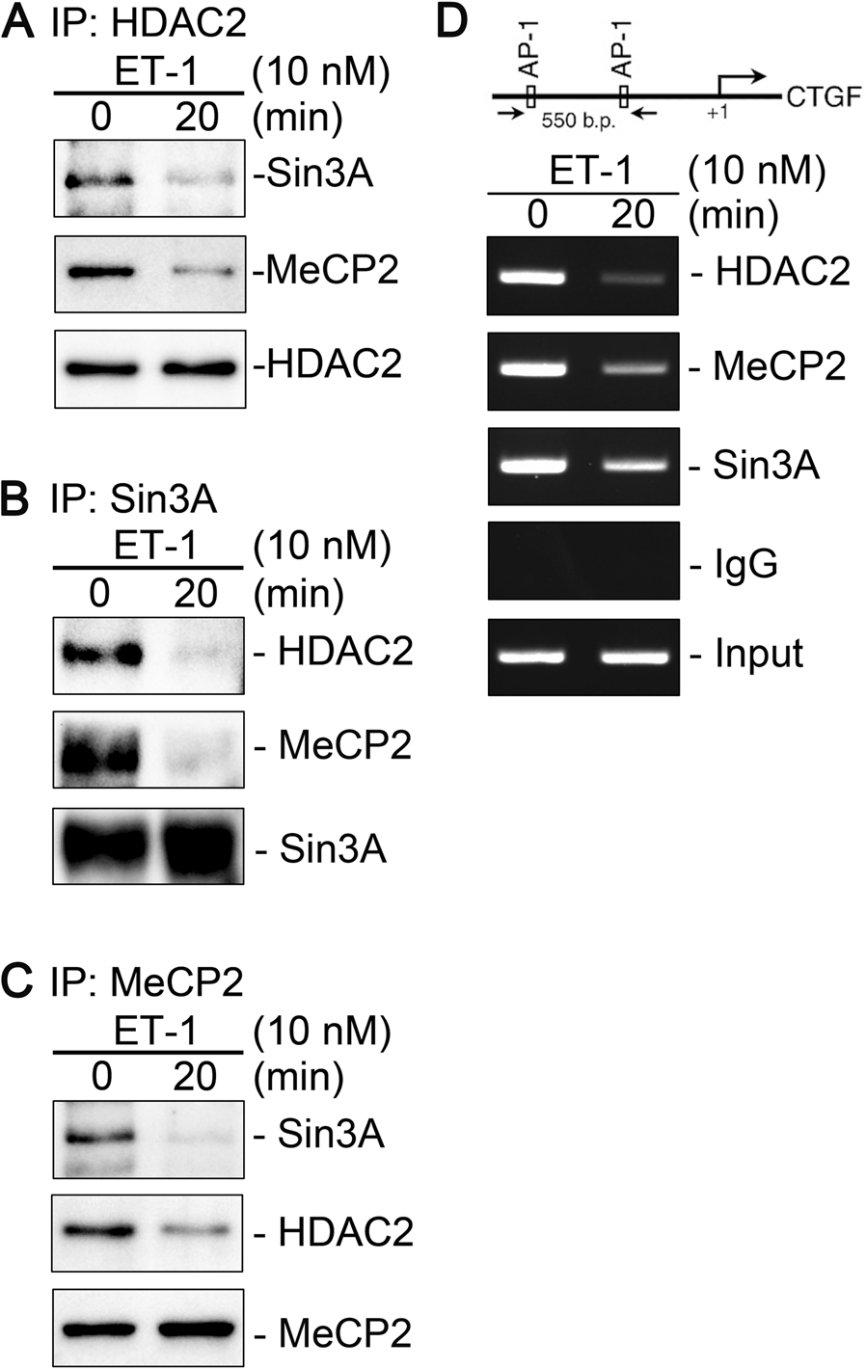
ET-1 treatment disrupted protein–protein interactions among HDAC2, Sin3A, and MeCP2 and induced dissociation of these corepressors from CTGF promoter region. Cells were stimulated with ET-1 for 20 min followed by the collection of lysates. Immunoprecipitation was then conducted with **A** HDAC2 (*n* = 5), **B** Sin3A (*n* = 5), or **C** MeCP2 (*n* = 4) antibodies. The protein–protein interaction among HDAC2, Sin3A, and MeCP2 was determined through Western blotting. **D** Schematic of the 550-bp ChIP primer located on the CTGF promoter. Cells were stimulated with ET-1 for 20 min, which was followed by ChIP assay. Nonimmune IgG was used as a negative control. Equal amounts of the soluble cross-linked chromatin present in each PCR were checked by the input (*n* = 5)

HDAC2 phosphorylation disrupts protein–protein interactions among corepressors HDAC2, Sin3A, and Yin Yang 1 [18]. Co-IP and ChIP assays were performed on ET-1-treated WI-38 cells to evaluate whether ET-1 disrupts the HDAC2/Sin3A/MeCP2 corepressor complex. The Co-IP assay indicated that ET-1 reduced protein–protein interactions among HDAC2, Sin3A, and MeCP2 (Fig. 6A–C). The ChIP assays revealed that ET-1 induced the dissociation of HDAC2, Sin3A, and MeCP2 from the CTGF promoter (Fig. 6D). Thus, ET-1 induced the disruption of the HDAC2/Sin3A/MeCP2 corepressor complex, which was followed by the dissociation of the complex from the CTGF promoter.

### HDAC2 was involved in the regulation of Sin3A- or MeCP2-suppressed AP-1 transcriptional activity in ET-1-treated WI-38 cells

**Fig. 7:**
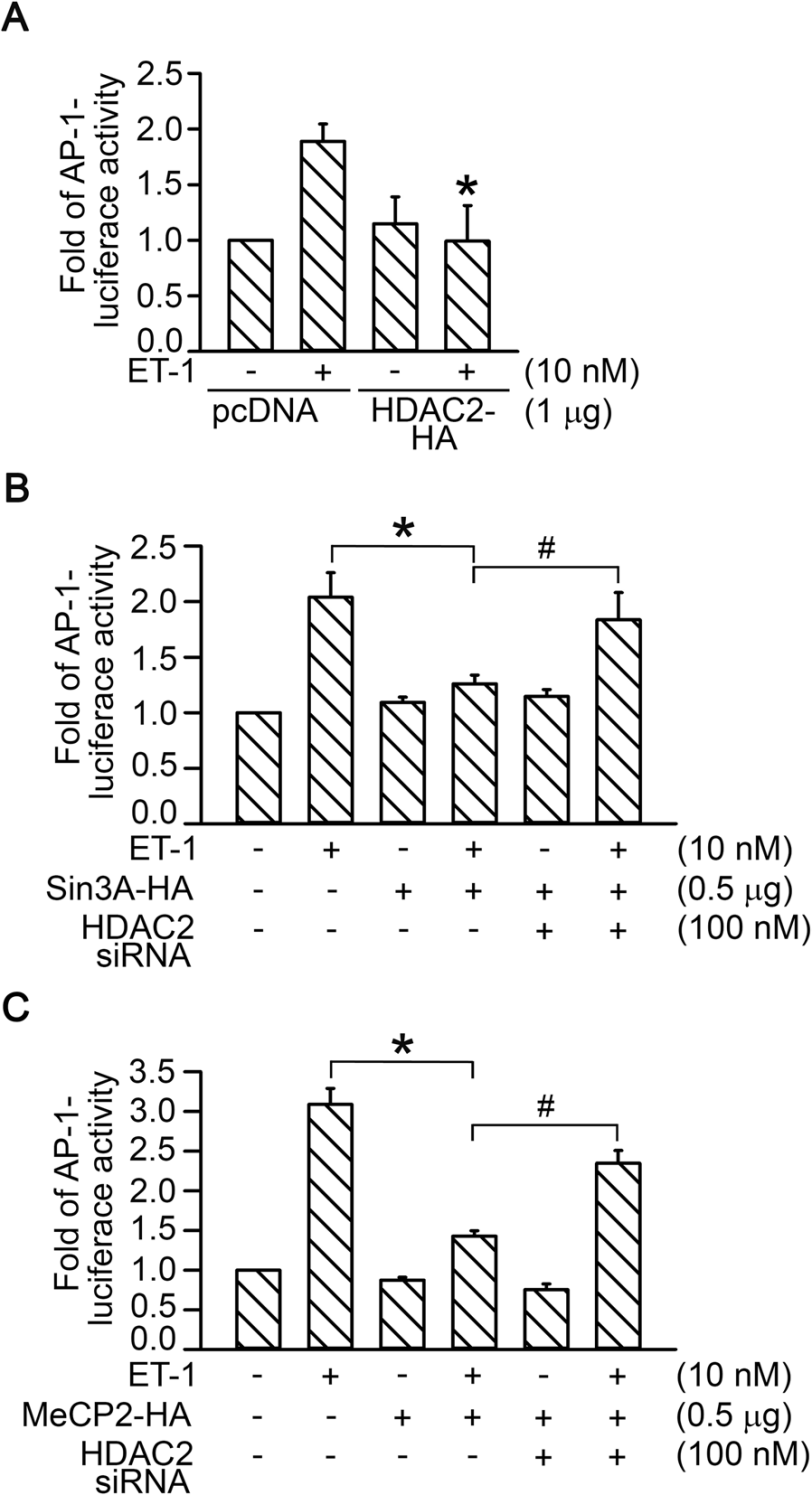
HDAC2 was involved in the regulation of Sin3A- or MeCP2-suppressed AP-1 transcriptional activity in ET-1-treated WI-38 cells. **A** Cells were transfected with HDAC2-HA (1 μg), AP-1-luciferase plasmid (1 μg), or pBK-CMV-Lac Z (0.1 μg) for 24 h and then stimulated with ET-1 for 16 h. Bars indicate values of mean ± SEM (*n* = 5). *p < 0.05 versus ET-1 stimulation. **B** Cells were transfected with AP-1-luciferase plasmid (1 μg) and pBK-CMV-Lac Z (0.1 μg) and then transfected with Sin3A-HA (0.5 μg) or co-transfected with Sin3A-HA (0.5 μg) and HDAC2 siRNA (100 nM) for 24 h. Cells were stimulated with ET-1 for 16 h. Luciferase activity was evaluated as described in “Materials and methods”. Bars indicate values of mean ± SEM (*n* = 5). *p < 0.05 versus ET-1-treated cells, ^#^*p* < 0.05 versus Sin3A-transfected cells with ET-1 stimulation. **C** Cells were transfected with AP-1-luciferase plasmid (1 μg) and pBK-CMV-Lac Z (0.1 μg) and then transfected with MeCP2-HA (0.5 μg) or co-transfected with MeCP2-HA (0.5 μg) and HDAC2 siRNA (100 nM) for 24 h. Cells were stimulated with ET-1 for 16 h. Luciferase activity was evaluated as described in “Materials and methods”. Bars indicate values of mean ± SEM (*n* = 5). *p < 0.05 versus ET-1-treated cells, ^#^*p* < 0.05 versus MeCP2-transfected cells with ET-1 stimulation

In our previous study, we revealed that ET-1 stimulated the production of CTGF through the ET_A_R/JNK/AP-1 signaling pathway [53]; ET-1 stimulation resulted in the recruitment of AP-1 to the CTGF promoter region and increased AP-1 transcriptional activity in WI-38 cells [53]. To elucidate the roles of HDAC2, Sin3A, and MeCP2 in regulating the transcriptional activity of AP-1, we examined the effect of HDAC2, Sin3A, or MeCP2 overexpression on ET-1-induced AP-1-luciferase activity. Transfection of HDAC2-HA plasmid (1 μg) into WI-38 cells attenuated ET-1-induced AP-1-luciferase activity by 100% ± 26% (*n* = 5; Fig. 7A). Transfection of Sin3A-HA plasmid (0.5 μg) attenuated ET-1-induced AP-1-luciferase activity by 75% ± 6% (*n* = 5; Fig. 7B). Furthermore, transfection of HDAC2 siRNA significantly reversed Sin3A-suppressed ET-1-induced AP-1-luciferase activity (*n* = 5; Fig. 7B). Transfection of MeCP2-HA plasmid (0.5 μg) suppressed ET-1-induced AP-1-luciferase activity by 79% ± 2% (*n* = 4; Fig. 7C). Furthermore, MeCP2-suppressed ET-1-induced AP-1-luciferase activity was significantly recovered by transfection of HDAC2 siRNA (*n* = 4; Fig. 7C). Thus, Sin3A- or MeCP2-regulated suppression of ET-1-stimulatd AP-1 transcriptional activity was dependent on the presence of HDAC2 in WI-38 cells.

### H3 acetylation was correlated with HDAC2 and Sin3A expression, but not MeCP2, in OVA-induced airway fibrosis

**Fig. 8:**
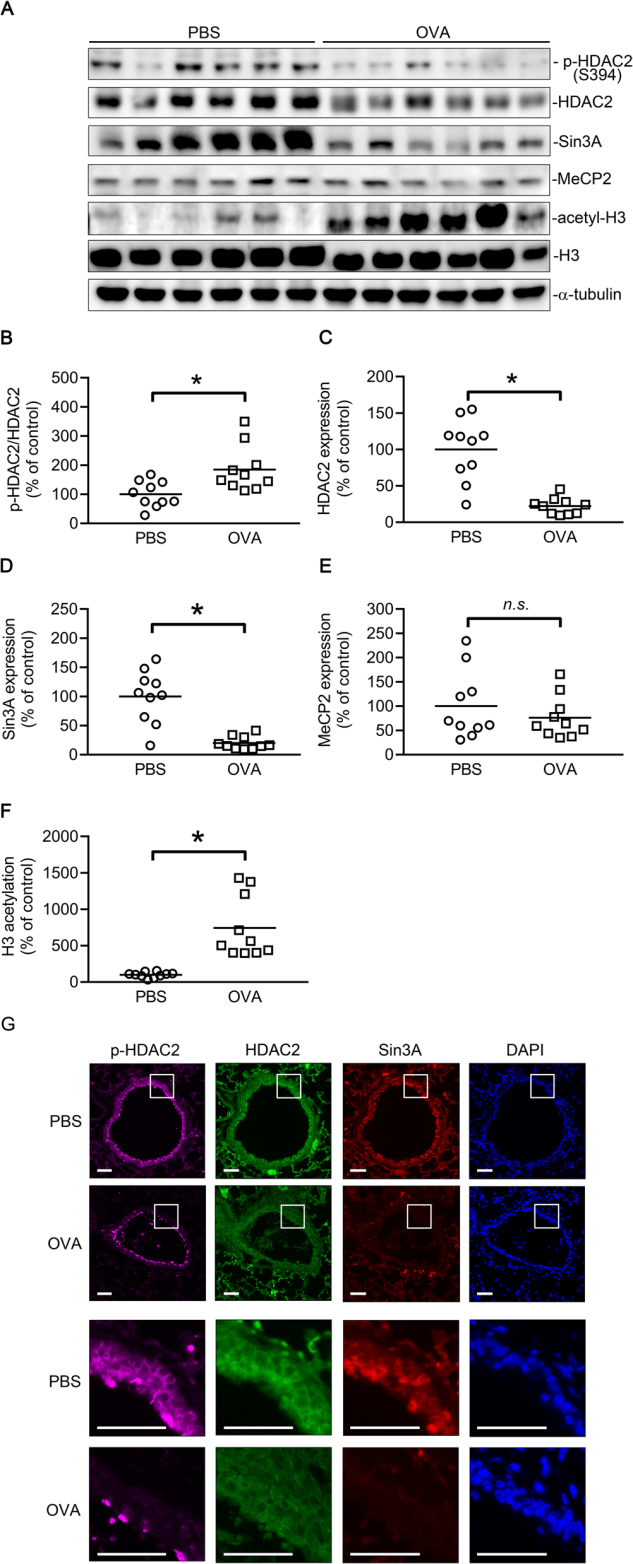
The level of phospho-HDAC2, HDAC2, Sin3A, and MeCP2 and acetylation of H3 in the lungs of mice with OVA-induced airway fibrosis. **A** The level of phospho-HDAC2, HDAC2, Sin3A, MeCP2, and acetylation of H3 in the lungs from PBS-treated or OVA-treated mice were evaluated through Western blotting. **B**–**F** Statistical analysis of protein levels. Horizontal lines indicate mean values, and each dot represents an individual mouse (*n* = 10). *p < 0.05 versus PBS group. **G** Paraffin sections of lung tissue from the PBS- or OVA-treated mice were stained for phospho-HDAC2 (purple), HDAC2 (green), Sin3A (red), and nuclei (blue). Bar, 50 μm

In a previous study, we demonstrated that the expressions of CTGF and fibrotic proteins, such as α-SMA and collagen, were increased in the lungs in an OVA-induced airway fibrosis model [10]. In this study, we evaluated the roles of HDAC2, Sin3A, and MeCP2 in OVA-induced airway fibrosis. The HDAC2, Sin3A, and MeCP2 protein levels, as well as HDAC2 phosphorylation and H3 acetylation, were analyzed using Western blotting. We observed that the ratio of phospho-HDAC2/HDAC2 was increased in the lung tissue of OVA-treated mice compared with the control group (*n* = 10; Fig. 8A, B). HDAC2 and Sin3A expression was lower (*n* = 10; Fig. 8A, C, D) in the lung tissue of OVA-treated mice compared with the control group. However, no significant difference was noted in the expression of MeCP2 (*n* = 10; Fig. 8A, E). H3 acetylation was markedly higher (*n* = 10; Fig. 8A, F) in the lung tissue of OVA-stimulated mice compared with the control group. Furthermore, using IF staining, the phosphorylation of HDAC2, and the expression of HDAC2 and Sin3A in the lung sections from the OVA-treated mice were examined. We found that the level of phospho-HDAC2, HDAC2, and Sin3A were reduced in the airway wall of OVA-treated mice compared with PBS-treated mice (Fig. 8G). Thus, an increase in H3 acetylation and a reduction in the protein levels in HDAC2 and Sin3A, but not MeCP2, might contribute to the pathogenesis of airway fibrosis.

## Discussion

ET-1 plays a vital role in lung fibrosis. The plasma concentration of ET-1 was reported to increase in patients with idiopathic pulmonary fibrosis [51]. ET-1 promotes fibroblast activation and differentiation, which lead to excessive ECM deposition [47]. Our previous study indicated that ET-1 stimulates the expression of CTGF through the ET_A_R/JNK/AP-1 signaling pathway and that CTGF is required for ET-1-stimulated myofibroblast differentiation [53]. Moreover, ET-1 activates p300 and HDAC7 to initiate AP-1 transcriptional activity and eventually promotes the expression of CTGF [22]. In this study, we found that ET-1 increased HDAC2 phosphorylation through MAPKs and then induced the dissociation of the HDAC2/Sin3A/MeCP2 corepressor complex from the CTGF promoter region, which in turn promoted histone H3 acetylation, thus increasing CTGF expression.

Histone acetylation results in the opening up of the chromatin structure, thereby allowing the binding of RNA polymerase II and transcriptions [23]. One study reported that histone acetylation might directly mediate inflammatory cytokine expression and that it participates in the progression of lung fibrosis [36]. Histone H3 acetylation was reported to contribute to the overexpression of profibrotic cytokine interleukin-6 in paraquat-induced pulmonary fibrosis [21]. HDAC2-containing complexes are critical regulators of histone acetylation [29]. In this study, HDAC2 overexpression inhibited ET-1-induced CTGF production and H3 acetylation in human lung fibroblasts. Transfection with HDAC2 siRNA enhanced ET-1-stimulated CTGF production. ET-1 stimulation reduced HDAC2 activity and increased H3 acetylation. Thus, HDAC2 negatively regulates CTGF expression through the suppression of H3 acetylation. Following ET-1 stimulation, the reduction of HDAC2 activity prompts an increase in H3 acetylation, which in turn promotes CTGF expression. On the other hand, our previous study found that activation of HDAC7 was involved in ET-1-stimulatd CTGF expression in human lung fibroblasts [22]. In this study, we found that overexpression of HDAC7 did not affect ET-1-stimulated H3 acetylation in WI-38 cells. Thus, ET-1-stimulated H3 acetylation was not regulated by HDAC7.

In one study, HDAC2 phosphorylation at serine sites S394, S422, and S424 negatively regulated its deacetylase activity; further, cigarette smoke attenuated the deacetylase activity of HDAC2 through casein kinase 2-mediated phosphorylation of HDAC2 [1]. Additionally, HDAC2 can possibly be phosphorylated by members of the MAPK family [44]. In this study, we demonstrated the following: ET-1 induces the increase in HDAC2 phosphorylation (S394), which was elevated in both nuclei and cytosol; pretreatment with JNK, ERK, or p38 inhibitors attenuates ET-1-induced HDAC2 phosphorylation and histone H3 acetylation; and the ET-1-induced reduction in HDAC2 activity is mitigated by JNK, ERK, or p38 inhibitors. Thus, ET-1 mediates the decrease in HDAC2 activity through MAPKs-mediated HDAC2 phosphorylation at S394. Indeed, as previous study suggested that S394 is responsible for the cardiac hypertrophy-associated activation of HDAC2 [17]. HDAC2 phosphorylation at S394 is important for the regulation of HDAC2 activity and its interactions with mSin3 and Mi2 [49]. On the other hand, a previous study suggested that all-trans retinoic acid-induced HDAC2 phosphorylation at S424 is mediated by JNK signaling [39]. However, the role of MAPKs in regulation of ET-1-stimulated HDAC2 phosphorylation at S422 and S424 still needs further study. In this study, we found that inhibitors of JNK, ERK, and p38 suppressed ET-1-stimulated HDAC2 phosphorylation at S394 by 77%, 89% and 96%, respectively. The p38 inhibitor possessed the highest level of inhibition rate. As a result, p38 may be the most important regulator in HDAC2 phosphorylation at S394.

HDAC2 is a prominent corepressor complex. These complexes also contain other specific proteins, such as Sin3A, crucial for HDAC2’s binding to DNA, recruitment to specific promoter sequences, and modulation of deacetylase activity [20]. A previous study suggested that loss of Sin3A drives alveolar type 2 cell dysfunction and progressive lung fibrosis in mice [57]. In addition, MeCP2 recruits HDAC2 and Sin3A to promote the deacetylation of histone tails, which results in gene silencing [28]. In this study, transfection of Sin3A or MeCP2 siRNA enhanced ET-1-stimulated CTGF production. Overexpression of Sin3A or MeCP2 attenuated ET-1-stimulated CTGF production. ET-1-induced H3 acetylation on CTGF promoter region was attenuated by overexpression of Sin3A or MeCP2. Sin3A- or MeCP2-suppressed ET-1-induced H3 acetylation were reversed by transfection with HDAC2 siRNA. These results suggest that HDAC2 is involved in the regulation of Sin3A- and MeCP2-suppressed H3 acetylation on CTGF promoter region, which results in inhibition of CTGF production in ET-1-treated cells. Furthermore, ET-1 induced the disruption of the HDAC2/Sin3A/MeCP2 corepressor complex and prompted the corepressor complex’s dissociation from the CTGF promoter region. Our previous study revealed that AP-1 participates in ET-1-stimulated CTGF expression [22]. In this study, we also found that overexpression of HDAC2, Sin3A, or MeCP2 attenuated ET-1-caused AP-1-luciferase activity. Moreover, Sin3A- or MeCP2-suppressed ET-1-stimulated AP-1-luciferase activity were reversed by transfection with HDAC2 siRNA. Thus, without stimulation, HDAC2 forms a corepressor complex with Sin3A and MeCP2 to inhibit H3 acetylation and AP-1 binding of CTGF promoter, thereby suppressing CTGF expression. Following ET-1 stimulation, the HDAC2/Sin3A/MeCP2 corepressor complex dissociates from the CTGF promoter region; subsequently, the increase in H3 acetylation allowing the binding of AP-1 to the CTGF promoter region, which results in an increase in AP-1 activity and stimulation of CTGF expression.

Airway remodeling is characterized by subepithelial fibrosis [4] and contributes to the progressive loss of lung function in asthma [41]. A previous study suggested that the presence of ET-1 is necessary to activate bronchial fibroblast proliferation and collagen synthesis in patients with asthma [16]. Moreover, ET-1 directs airway remodeling and hyper-reactivity in a murine asthma model [19]. In addition, ET-1 is increased in the bronchoalveolar lavage fluid from OVA-challenged mice and is involved in the process of subepithelial fibrosis [7]. In our previous studies, the expressions of CTGF, α-SMA, and collagen were increased in the lungs in an OVA-induced airway fibrosis model [10], and the HDAC7 overexpression might play an important role in the pathogenesis of OVA-induced airway fibrosis [22]. A related study suggested that an increase in H3 acetylation in lung tissue was associated with asthma pathogenesis [45]. In this study, we also found that histone H3 acetylation considerably increased in lung tissue of OVA-stimulated mice. Thus, the increase in H3 acetylation might be involved in the pathogenesis of OVA-induced airway fibrosis. HDAC2-containing complexes are vital mediators of histone deacetylation in vivo [29]. In another study, HDAC2 formed a corepressor complex with MeCP2 and Sin3A to promote the deacetylation of histone tails, which resulted in gene silencing [28]. In inflammatory lung diseases, such as COPD and severe asthma, a reduction in the HDAC2 protein level or HDAC2 activity is commonly observed [2]. One study reported that HDAC2 activation could prevent airway remodeling through the suppression of airway inflammation and the modulation of fibroblast activation in COPD [33]. In the present study, HDAC2 phosphorylation and protein level of HDAC2 and Sin3A significantly decreased in the lung of OVA-stimulated mice. Although the level of phospho-HDAC2 and HDAC2 were both decreased, the ratio of phospho-HDAC2/HDAC2 was significantly increased in the lung of OVA-stimulated mice. Moreover, IF staining also showed that the level of HDAC2 and Sin3A were reduced in the airway wall of OVA-treated mice compared with control group. These results suggest that HDAC2 phosphorylation and the loss of HDAC2 and Sin3A levels might be involved in the pathogenesis of OVA-caused airway fibrosis. On the other hand, MeCP2 is instrumental to the deacetylation activity of HDACs. MeCP2 recruits HDACs and Sin3 to methylated DNA and suppresses gene expression [27]. However, we observed that the MeCP2 protein level was unchanged in the lung tissue of an OVA-induced airway fibrosis model. Therefore, MeCP2 protein level may play less of a role than HDAC2 and Sin3A in the pathogenesis of OVA-induced airway fibrosis.

## Conclusions

**Fig. 9:**
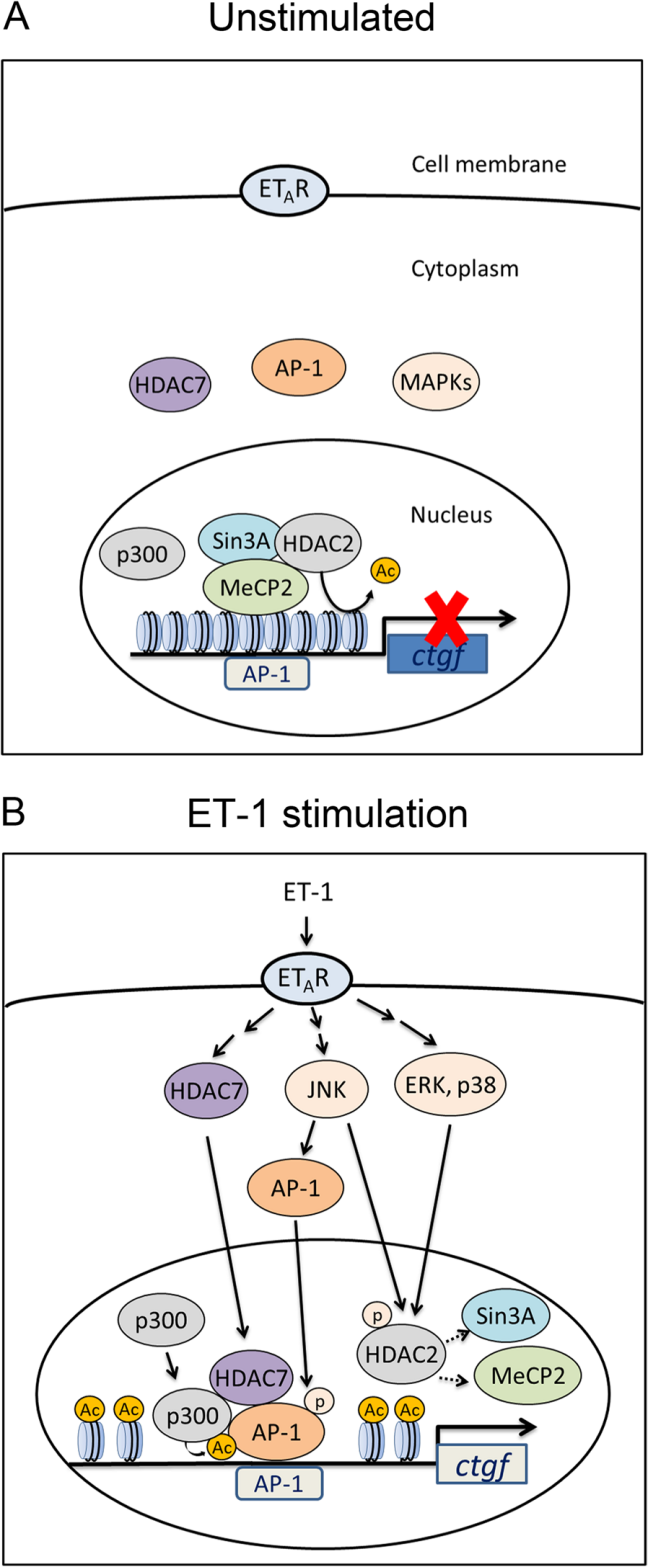
Schematic of how ET-1 signal transduction promotes CTGF production. **A** Without stimulation, HDAC2 inhibits CTGF production through the formation of a corepressor complex with Sin3A and MeCP2, thereby suppressing H3 acetylation on the CTGF promoter. **B** With ET-1 stimulation, MAPKs mediate HDAC2 phosphorylation, which is followed by the disruption of the corepressor complex and acetylation of H3 in the CTGF promoter region; this prompts the opening up of the chromatin structure to allow binding of AP-1. By contrast, ET-1 recruits AP-1 to the CTGF promoter region through JNK-mediated AP-1 phosphorylation and HDAC7/p300/AP-1 transcriptional complex formation, which in turn induces CTGF expression

Without stimulation, the HDAC2/Sin3A/MeCP2 corepressor complex inhibited CTGF expression through H3 deacetylation in the CTGF promoter region (Fig. 9A). HDAC2, Sin3A, and MeCP2 served as the corepressors of CTGF expression. With ET-1 stimulation, HDAC2 activity was reduced through MAPK-mediated HDAC2 phosphorylation followed by dissociation of the HDAC2 corepressor complex from the CTGF promoter as well as H3 acetylation; subsequently, the chromatin structure opened up to allow binding of AP-1, which in turn caused CTGF production (Fig. 9B). The present results, together with those of our previous reports, suggest the following: ET-1 stimulation induces AP-1 activation through the ET_A_R/JNK pathway [53] as well as the formation of the HDAC7/p300/AP-1 transcriptional complex [22]; subsequently, transcriptional complex is recruited to the CTGF promoter, thereby stimulating CTGF production (Fig. 9B). Our results highlight the vital role of the HDAC2/Sin3A/MeCP2 corepressor complex in preventing CTGF production in human lung fibroblasts. Further, pathologic loss of HDAC2 and Sin3A rather than MeCP2 may be a key determinant in the pathogenesis of airway fibrosis.

## Data Availability

The datasets used and/or analysed during the current study are available from the corresponding author on reasonable request.

## Abbreviations

AP-1: Activator protein-1
ChIP: Chromatin immunoprecipitation
COPD: Chronic obstructive pulmonary disease
CTGF: Connective tissue growth factor
ECM: Extracellular matrix
ERK: Extracellular signal-regulated kinase
ET: Endothelin
ET_A_R: ETA receptor
FBS: Fetal bovine serum
HDAC: Histone deacetylase
IF: Immunofluorescence
JNK: C-Jun N-terminal kinases
MAPK: Mitogen-activated protein kinase
MECP: Methyl-CpG-binding protein
MEM: Minimum essential medium
OVA: Ovalbumin
PBS: Phosphate-buffered saline
PCR: Polymerase chain reaction
siRNA: Small interfering RNA
TGF-β: Transforming growth factor-β
